# Early short-term PXT3003 combinational therapy delays disease onset in a transgenic rat model of Charcot-Marie-Tooth disease 1A (CMT1A)

**DOI:** 10.1371/journal.pone.0209752

**Published:** 2019-01-16

**Authors:** Thomas Prukop, Jan Stenzel, Stephanie Wernick, Theresa Kungl, Magdalena Mroczek, Julia Adam, David Ewers, Serguei Nabirotchkin, Klaus-Armin Nave, Rodolphe Hajj, Daniel Cohen, Michael W. Sereda

**Affiliations:** 1 Max-Planck-Institute of Experimental Medicine, Department of Neurogenetics, Göttingen, Germany; 2 University Medical Center Göttingen, Institute of Clinical Pharmacology, Göttingen, Germany; 3 Pharnext, Issy-Les-Moulineaux, France; 4 University Medical Center Göttingen, Department of Clinical Neurophysiology, Göttingen, Germany; Yale University School of Medicine, UNITED STATES

## Abstract

The most common type of Charcot-Marie-Tooth disease is caused by a duplication of *PMP22* leading to dysmyelination, axonal loss and progressive muscle weakness (CMT1A). Currently, no approved therapy is available for CMT1A patients. A novel polytherapeutic proof-of-principle approach using PXT3003, a low-dose combination of baclofen, naltrexone and sorbitol, slowed disease progression after long-term dosing in adult *Pmp22* transgenic rats, a known animal model of CMT1A. Here, we report an early postnatal, short-term treatment with PXT3003 in CMT1A rats that delays disease onset into adulthood. CMT1A rats were treated from postnatal day 6 to 18 with PXT3003. Behavioural, electrophysiological, histological and molecular analyses were performed until 12 weeks of age. Daily oral treatment for approximately 2 weeks ameliorated motor deficits of CMT1A rats reaching wildtype levels. Histologically, PXT3003 corrected the disturbed axon calibre distribution with a shift towards large motor axons. Despite dramatic clinical amelioration, only distal motor latencies were improved and correlated with phenotype performance. On the molecular level, PXT3003 reduced *Pmp22* mRNA overexpression and improved the misbalanced downstream PI3K-AKT / MEK-ERK signalling pathway. The improved differentiation status of Schwann cells may have enabled better long-term axonal support function. We conclude that short-term treatment with PXT3003 during early development may partially prevent the clinical and molecular manifestations of CMT1A. Since PXT3003 has a strong safety profile and is currently undergoing a phase III trial in CMT1A patients, our results suggest that PXT3003 therapy may be a *bona fide* translatable therapy option for children and young adolescent patients suffering from CMT1A.

## Introduction

Charcot-Marie-Tooth disease (CMT) is the most common inherited peripheral neuropathy with a prevalence over one in 2500 [1,2]. With Next Generation sequencing over 90 genes were linked to CMT [3,4], of which the most common type (CMT1A, over 39%, [5,6] is caused by a duplication of the gene encoding for the peripheral myelin protein of 22 kDa (*PMP22*) [7–9]. Although dysmyelination and onion bulb formation are prominent histological hallmarks, the progressive clinical phenotype is determined by the degree of axonal loss which cause progressive muscle weakness and sensory deficits [10–15]. Although disease variability is high, walking disabilities, foot deformities and electrophysiological abnormalities are already present in childhood and can lead to severe disabilities throughout life-span [16–18]. Recently, young CMT1A patients were found to progress at a rate of 14% using the CMT Pediatric Scale (CMTPedS) which is a sensitive outcome measure for clincal trials [18]. Despite several promising trials in animal models, no causal treatment is available for any form of CMT yet [14,19]. Neither trials of exercise and orthosis, nor pharmacological such as oral administration of ascorbic acid showed beneficial effects in patients with CMT1A [17,20–23]. The early onset of the disease raises the question of an effective, in best-case a preventive therapy option for young CMT1A patients.

PXT3003 is a combination of low-dose baclofen, naltrexone and sorbitol. The single composite drugs were identified by using a systems biology approach in order to target G-coupled receptor signalling pathways that were predicted to lower *Pmp22* mRNA expression, but also pathways important for myelination and axonal integrity. PXT3003 was reported to reduce *Pmp22* mRNA expression in vitro and slow disease progression in adult phenotypically affected CMT1A rats after chronic long-term dosing [24]. Importantly, due to synergistic action by each of the single drugs, PXT3003 can be applied at an approximately 10-fold lower dose than the approved dose of single drugs. Tolerability and safety were proven in an exploratory clinical phase II study in CMT1A patients [25,26]. Currently, the efficacy of PXT3003 is being investigated in a confirmatory randomised, double-blind, placebo-controlled phase III study (*ClinicalTrials*.*gov identifier NCT03023540*).

Increased *Pmp22* levels in Schwann cells induce an early postnatal differentiation defect characterized by the aberrant expression of genes characteristic for immature Schwann cells such as *Sox2* and *c-Jun* [27,28]. While CMT1A is caused by a primary defect in Schwann cells, the consequent dysmyelination and lack of trophic support finally leads to axonal dysfunction and loss. Hence, promoting Schwann cell differentiation may be one possible approach—beyond *Pmp22* mRNA regulation itself—to prevent dysmyelination and the resulting axonal degeneration leading to clinical symptoms. We previously demonstrated an early reduction of the phosphatidylinositol 4,5-bisphosphate 3-kinase (PI3K)-AKT murine thymoma viral oncogene homolog 1 (AKT) signaling in CMT1A rat sciatic nerves during early postnatal development (postnatal day 1, P1) which was followed by an increase of the mitogen-activated protein kinase 1 (MEK)–mitogen-activated protein kinase (ERK) at P6. PI3K-AKT and MEK-ERK signalling are two opposing pathways regulating Schwann cell differentiation. The binding of axonally derived neuregulin-1 to ErbB receptors in the Schwann cell plasma membrane activates the PI3K-AKT signalling cascade, thereby regulating myelin sheath thickness in the PNS [29]. In a proof-of-principle approach, we applied the soluble EGF-like domain of recombinant human neuregulin-1 (rhNRG1) early postnatally over short-term during the critical time window of Schwann cell differentiation in CMT1A rats. rhNRG1 treatment increased PI3K-AKT and decreased MEK-ERK signalling which drove *Pmp22* transgenic Schwann cells towards differentiation and preserved peripheral nerve axons. Consecutively, muscle strength was restored until adulthood [27]. These observations highlighted the importance of an early therapeutic window for future drug therapy regimes in CMT1A patients.

Hence, in the present study, we tested the hypothesis that PXT3003 treatment downregulates *Pmp22* during the critical time window of Schwann cell differentiation and can overcome the known impaired peripheral nerve development in CMT1A. This, in turn, may cause sustained axonal support and amelioration of the clinical phenotype into adulthood.

## Materials and methods

### Animals and husbandry

Male *Pmp22* transgenic Sprague-Dawley rats (CMT1A rats, [30]) and corresponding male wildtype controls were used. All rats were housed in a climate-controlled environment on a 12h light/dark cycle with free access to water and sorbitol-free food (V1325-318, SNIFF, Germany).

### Genotyping

Animals were genotyped by standardized methods based on the polymerase chain reaction (PCR) as previously described [27]. After local anaesthesia with lidocaine gel 2%, a tail biopsy was taken from 2 days old rats, and the paws were tattooed for identification. Isolation of genomic DNA from tail biopsies was performed according to standard manufacturer’s instructions (Qiagen, Germany).

### Test items, vehicle, dosages and application

PXT3003 is a mix in a fixed ratio of baclofen, naltrexone hydrochloride and D-sorbitol (Sigma-Aldrich, Germany) dissolved in phosphate buffer at pH = 5.4, and stored between +2°C and +8°C protected from light for the maximum of 1 week. Phosphate buffer alone was used as placebo vehicle for wildtype and CMT1A controls. Three increasing dosages of PXT3003 were applied to CMT1A rats by daily oral gavage with baclofen 15 μg/kg/day, naltrexone hydrochloride 1.75 μg/kg/day and D-sorbitol 0.525 mg/kg/day (called PXT3003-1 in the following), baclofen 30 μg/kg/day, naltrexone hydrochloride 3.5 μg/kg/day and D-sorbitol 1.05 mg/kg/day (PXT3003-2), and baclofen 60 μg/kg/day, naltrexone hydrochloride 7 μg/kg/day and D-sorbitol 2.1 mg/kg/day (PXT3003-3).

### Sample size, group allocation, study regimen and euthanasia

Known treatment effects from our former studies in CMT1A rats were used for the sample size calculation. Group allocations were performed by random and stratified for the genotype, weight and litter allocation. Early short-term treatment took place from postnatal day 6 until 18 followed by long-term observational phenotyping at postnatal weeks 3, 9 and 12. At study end, rats were anesthetised for additional electrophysiological measures. Anaesthesia was applied as 100 mg/kg body weight ketamine (Ketanest) and 20 mg/kg xylazine (Rompun) in a 1:1 ratio. CO_2_ inhalation without reawakening from the anaesthesia was used prior to tissue collections for histology, RNA and protein analysis.

### Phenotyping

All phenotype tests were performed blinded by the same investigator on the same time every day. Motor performance was measured by grip strength investigation for the forelimbs and hindlimbs using a digital newton meter (Model 708, Erichsen, Germany). Rats were motivated to grip a horizontal T-bar with their forelimbs and the investigator pulled them horizontally down until the rats lost grip. Hind limb grip strength was measured by supporting the fore limbs and pulling the animal’s tail toward the horizontal bar. Each time the maximum force exerted onto the T-bar before the animals lost grip was recorded. Measures were repeated at least 5 times per limb and the means were calculated. The inclined plane test was used to measure the motor coordination by using a plexiglas plane adjusted to an angle of 25°. Rats were placed on the inclined plane in the up-headed position as published by Rivlin and colleagues [31]. Measures were repeated at least 2 times per animal and the means were calculated. The performance was evaluated by 4 different scores: no slide = 0, sliding of one or two paws = 1, sliding of all four paws = 2, sliding to the very bottom of the plane = 3. Compound muscle action potentials (CMAP), distal motor latencies (DML) and nerve conduction velocities (NCV) were measured at the tail of anesthetized rats (Model Neuroscreen, Jaeger-Toennies, Germany). The tail was placed into an oil bath (37°C) to keep the tail′s temperature constant for the measurements. Stimulation was performed with increasing voltage of 0.1ms duration until supramaximal stimulation. Maximum CMAPs were calculated peak to peak, DML as interval between the stimulation and the observed response and nerve conduction velocity from the distance between proximal and distal stimulation.

### Histology

Two peripheral nerves (tibial and sciatic nerve) were removed and fixed in 4% paraformaldehyde and 2.5% glutaraldehyde in phosphate buffer, then embedded in epoxy resin and cut into 0.5 μm thick cross sections using a microtome (Leica, Germany). After staining with methylene blue / Azur II, the total number of myelinated axons per nerve was counted. In more detail, myelin sheath thickness (g-ratio = axon circumference divided by circumference of the axon plus myelin sheath) and axon diameter of 150 axons nerve were analysed which were randomly distributed throughout the entire. This analysis was performed on light microscopic images by a blinded investigator using open-source ImageJ software.

### RNA and protein expression

Two peripheral nerves (sciatic nerve and brachial plexus) were frozen in liquid nitrogen and stored at -80°C until use. For RNA and protein isolation samples were homogenized in 80% sucrose buffer containing protease inhibitor and phosphatase inhibitor according to manufacturer′s instructions (Roche, Germany). The lysate was added to RLT buffer and RNA isolation followed manufacturer’s instructions (Qiagen, Germany). For quantitative reverse transcriptase PCR (RT-PCR) analysis, cDNA was synthesized from total RNA using random nonamer primers and Superscript III reverse transcriptase (ThermoFischer, Germany). SYBR Green RT-PCR master mix (Qiagen, Germany) was prepared to a final reaction volume of 10 μl. The RT-PCR followed a two-step protocol (Model LC480, Roche,Germany), and quantitation of PCR product was performed using the comparative ΔΔCt method. Each sample was measured in at least three replicates and averaged. The genes of interest and housekeeping genes used for normalisation are indicated in the corresponding figure legends. In case of total *Pmp22*, primers were designed to quantify endogenous and transgene-derived gene expression containing Schwann cell specific exon 1A and ubiquitously expressed exon 1B [32]. *Pmp22* splice variant primers were adapted according to Visigalli et colleagues to cover exon 1B containing splice variants 2 and 6, which however both were shown to be expressed in peripheral nerves [33]. Primer sequences were as follows: total *Pmp22* sense 5`-TGTACCACATCCGCCTTGG-3`and antisense 5`-GAGCTGGCAGAAGAACAGGAAC-3`, *Pmp22* splice variant sense 5`-GCTGTCCCTTTGAACTGAAA-3`and antisense 5`-GAACAGGATCCCCAACAAGAGTAG-3`, *cJun* sense 5`-CCTTCTACGACGATGCCCTC-3`and antisense 5`-GGTTCAAGGTCATGCTCTGTTT-3`, *Sox2* sense 5`-TCCAAAAACTAATCACAACAATCG-3`and antisense 5`-GAAGTGCAATTGGGATGAAAA-3`, *Ctsa* sense 5`-TTACAGAGCACGGTCCCTTC-3` and antisense 5`-ATATACAGCATGTTGGCAATCAG-3`, *Hmgcr* sense 5ʹ-CAACCTTCTACCTCAGCAAGC-3ʹ and antisense 5'-CACAGTGCCACACACAATTCG-3'; *Prx* sense 5'-GAGCCTCAGTTTGCAGGAAG-3' and antisense 5'-TTGTAGGGCTCGGCACAT-3'; *Mpz* sense 5`-GTCCAGTGAATGGGTCTCAGATG-3`and antisense 5`-CTTGGCATAGTGGAAGATTGAAA-3`, *Cyclophilin-A* sense 5`-AGCACTGGGGAGAAAGGATT-3`and antisense 5`-AGCCACTCAGTCTTGGCAGT-3`, and *Rplp0* sense 5`-GATGCCCAGGGAAGACAG-3`and antisense 5`-CACAATGAAGCATTTTGGGTAG-3`.

Remaining lysate was used for Western Blot analyses of previously reported misbalanced phosphorylated / non-phosphorylated PI3K-AKT and MEK-ERK as a *Pmp22* downstream signalling pathway in CMT1A [27]. Protein concentration was determined by the Bradford assay and equal amounts were denatured in Laemmli buffer containing 10% β-mercaptoethanol. Gel electrophoresis was carried out for 90 minutes at 100V (BioRad, Germany). Transfer was carried out for 1 hour at 350 mA (BioRad, Germany). Relative protein amounts were determined via staining the membranes with fast green (Sigma, Germany) and using a fluorescent imager (Intas Science Imaging, Germany). Membranes were then washed briefly in washing buffer and then transferred to blocking buffer for 1 hour. Western blots were incubated overnight with primary antibodies against p-AKT, AKT, p-MAPK, MAPK (all polyclonal rabbit; 1:1,000, Cell Signaling: pAKT No. 3787S, batch No. 8; AKT No. 4691S, batch No. 20; MAPK No. 4695S, batch No. 21; pMAPK No. 9101S, batch No. 30), and 30 min with a peroxidase conjugated secondary antibody (AffiniPure goat anti-rabbit IgG (H+L); 1:1000; Jackson Immuno Research Laboratories, USA). Quantification was performed using Western Lightning Plus-ECL, Enhanced Chemiluminescence Substrate (Perkin Elmer) and a luminescence imager (Intas Science Imaging, Germany).

### Statistics

GraphPad Prism software was used for all analysis. Data is presented as means ± the standard deviation. Significance was determined by applying the unpaired students T-Test; ns = not significant, * = p<0.05, **p<0.01 and *** = p<0.001.

### Study approval

All experimental procedures involving animals were discussed by an in-house review committee considering the sense and need of the study incl. animal welfare, and further approved by the Niedersächsisches Landesamt für Verbraucherschutz und Lebensmittelsicherheit (LAVES) by approval number 33.19-42502-04-15/1748 prior to study start.

## Results

**Fig 1:**
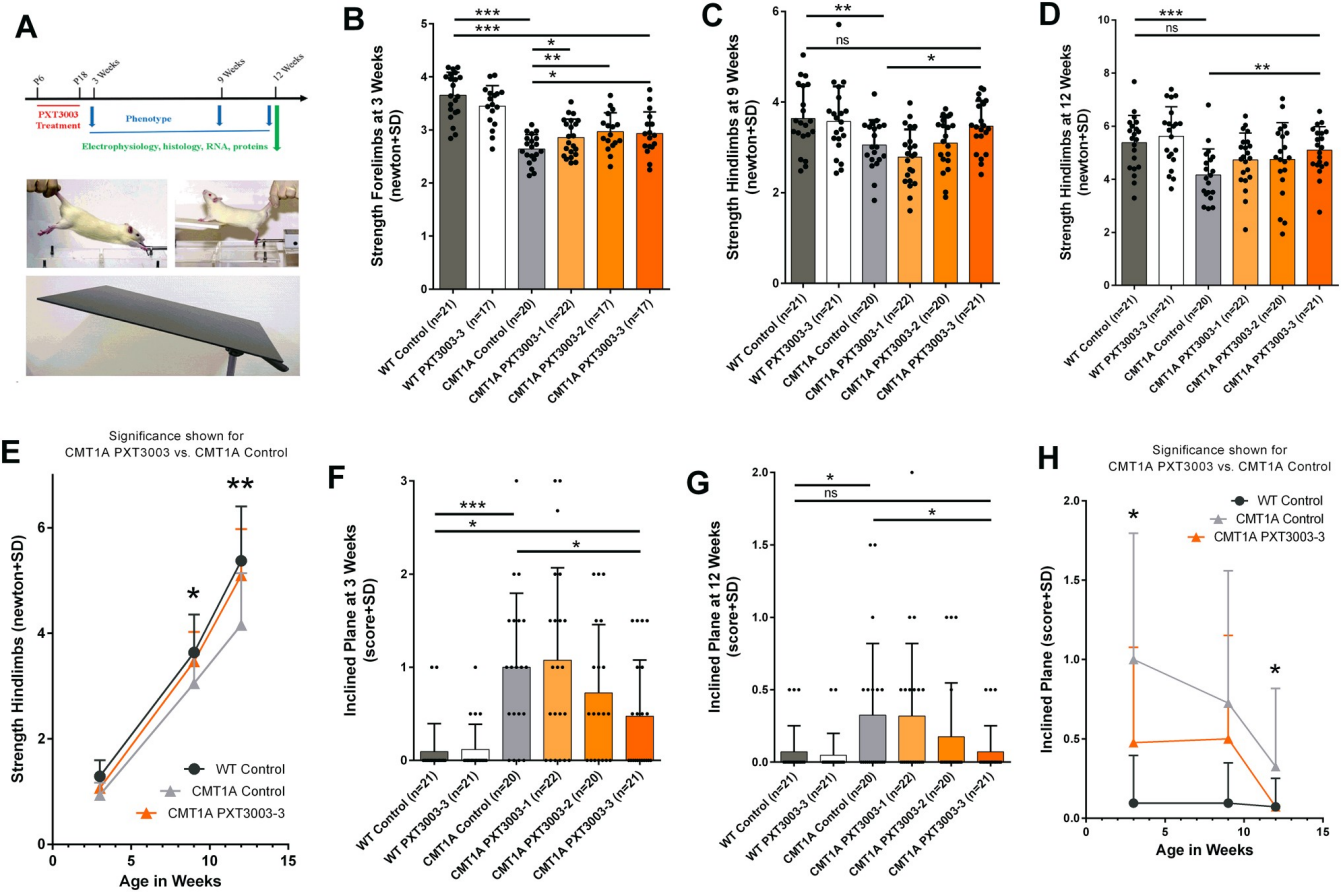
Long-lasting restoration of phenotype deficits after early short-term PXT3003 therapy. Early short-term therapy regimen followed by a long-term observation of motor and sensory, histological and molecular effects (A). Early postnatally PXT3003 application dose-dependently improved the grip strength impairment at the forelimbs after the treatment phase in 3 weeks aged CMT1A rats (B; WT controls 3.66±0.09, CMT1A controls 2.64±0.06, CMT PXT3003-3 2.93±0.10). First grip strength improvement was observed at the hindlimbs in 9 weeks aged CMT1A rats (C; WT controls 3.64±0.16, CMT1A controls 3.06±0.12, CMT PXT3003-3 3.47±0.12) lasting until 12 weeks (D; WT controls 5.38±0.23, CMT1A controls 4.16±0.22, CMT PXT3003-3 5.10±0.19). PXT3003 long-term effects increased over time reaching wildtype levels in the long-term observation (E). PXT3003 early short-term therapy improved motor deficits on the inclined plane in 3 (F; WT controls 0.10±0.07, CMT1A controls 1.00±0.18, CMT PXT3003-3 0.48±0.13) and 12 weeks aged CMT1A rats (G; WT controls 0.07±0.04, CMT1A controls 0.33±0.11, CMT PXT3003-3 0.07±0.04). Although some training effects were observed in the inclined plane behaviour in CMT1A controls, PXT3003 effects increased over time and reached wildtype levels at the study end (H). (ns = not significant, * = p<0.05, **p<0.01 and *** = p<0.001).

PXT3003 treatment in CMT1A rats was performed over 13 days in three increasing dosages (PXT3003-1/-2/-3) early short-term from postnatal day 6 until 18 followed by long-term observational phenotyping at postnatal week 3, 9 and 12. Also, wildtype rats received PXT3003 for adverse effect investigations, and CMT1A and wildtype rats treated with placebo served as controls. At the end of the study electrophysiological measurements, histological evaluation and mRNA and protein expression analysis were conducted in peripheral nerves (Fig 1A).

### Increasing long-term phenotype effects after early short-term PXT3003 therapy

At treatment start, i.e. before the manifestation of obvious phenotype deficits, body weights did not differ between 6 days aged wildtype and CMT1A controls, and all CMT1A PXT3003 groups showed comparable body weights representing a well-balanced randomisation (S1A Fig). Throughout the long-term observational study duration until postnatal week 12, CMT1A controls showed a reduced body weight (S1B Fig), weaker muscle strength and impaired motor coordination compared to wildtype controls. Generally, no adverse effects were observed in PXT3003 treated wildtypes regarding the body weight (S1B Fig), muscle strength (Fig 1B–1D) and motor coordination (Fig 1F and 1G).

In the grip strength analysis, we observed a significant therapeutic improvement of the forelimb strength once early in 3 weeks aged PXT3003 short-term treated CMT1A rats (i.e 3 days after treatment cessation) for all PXT3003 dosages compared to CMT1A controls (Fig 1B and S1C Fig). In contrast, at this time point, hind limb muscle strength only showed a dose-dependent trend in PXT3003 short-term treated CMT1A rats (without reaching significance), possibly owing to the longer axons innervating muscles of the hindlimb in line with previous experimental trials in CMT1A rats [32,34] (Fig 1E). However, at 9 weeks of age PXT3003-3 short-term treated CMT1A rats showed increased hind limb muscle strength, when compared to CMT1A controls, reaching wildtype levels (Fig 1C). This treatment effect even increased until the study end at postnatal week 12 (Fig 1D and 1E).

Motor performance was also measured using the inclined plane test [31]. At postnatal week 3, we already observed a dose-dependent significant improvement of motor coordination in PXT3003 short-term treated CMT1A rats when compared to littermate controls (Fig 1F). 9 weeks later, the short-term treatment effect on motor coordination further increased in 12 week aged CMT1A rats treated with PXT3003-3 to reach wildtype levels (Fig 1G and 1H). Despite training effects observed in CMT1A rats, placebo-treated CMT1A controls did not reach wildtype levels at study end in contrast to PXT3003-treated CMT1A rats (Fig 1G). Wildtype rats did not show relevant phenotypical deficits in the inclined plane test throughout the study, and therefore no training effect could be observed (Fig 1H).

### Improved distal motor latencies at end of trial

**Fig 2:**
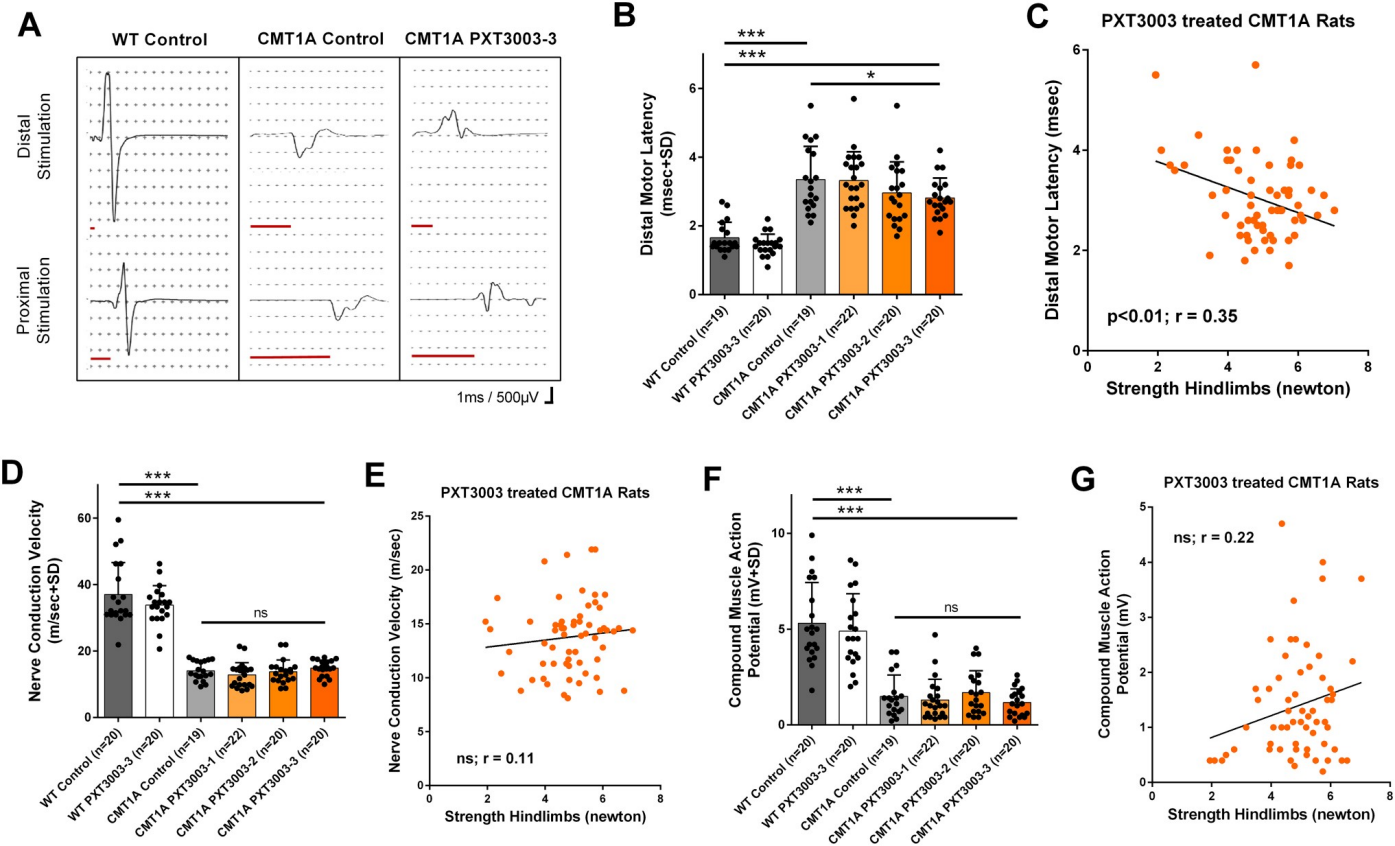
Improved motor latency correlating with the phenotype at study end. Electrophysiological recordings including motor latency (red bar), nerve conduction velocity (NCV) and compound muscle potential (CMAP) analysis were performed as shown for representative measures (A). CMT1A controls showed prolonged motor latencies, which were dose-dependently improved by PXT3003 early short-term treatment (B; WT controls 1.66±0.10, CMT1A controls 3.35±0.22, CMT PXT3003-3 2.82±0.13). In long-term observation, the motor latency improvement correlated with the restored grip strength at the hindlimbs including all PXT3003 treated CMT1A rats (C). NCV and CMAP recordings were not affected by PXT3003 in long-term (D and F; WT controls 37.02±2.16, CMT1A controls 14.02±0,67, CMT PXT3003-3 14.86±0.53 and WT controls 5.31±0.47, CMT1A controls 1.49±0.25, CMT PXT3003-3 1.18±0.16, respectively), and did not correlate with the hindlimb grip strength of all PXT3003-treated CMT1A rats (E and G). (ns = not significant, * = p<0.05 and *** = p<0.001).

At the end of the long-term observational period (at 12 weeks of age), we detected significant prolongation of the motor latencies, reduction of the nerve conduction velocity (NCV) and the compound motor action potential (CMAP) in CMT1A controls compared to the wildtype controls (representative recordings shown in Fig 2A). Early short-term PXT3003 treatment in CMT1A rats dose-dependently improved distal motor latencies reaching significance for PXT3003-3 compared to CMT1A controls (Fig 2B). However, we did not detect significant therapy effects on NCV and CMAP recordings after PXT3003 short-term treatment in CMT1A rats compared to corresponding CMT1A controls (Fig 2D and 2F). In line with these results, distal motor latencies significantly correlated with hind limb muscle strengths in all 12 weeks aged PXT3003 treated CMT1A rats (Fig 2C), whereas NCV and CMAP recordings did not (Fig 2E and 2G).

### Increased number of larger-calibre axons after PXT3003 therapy

**Fig 3:**
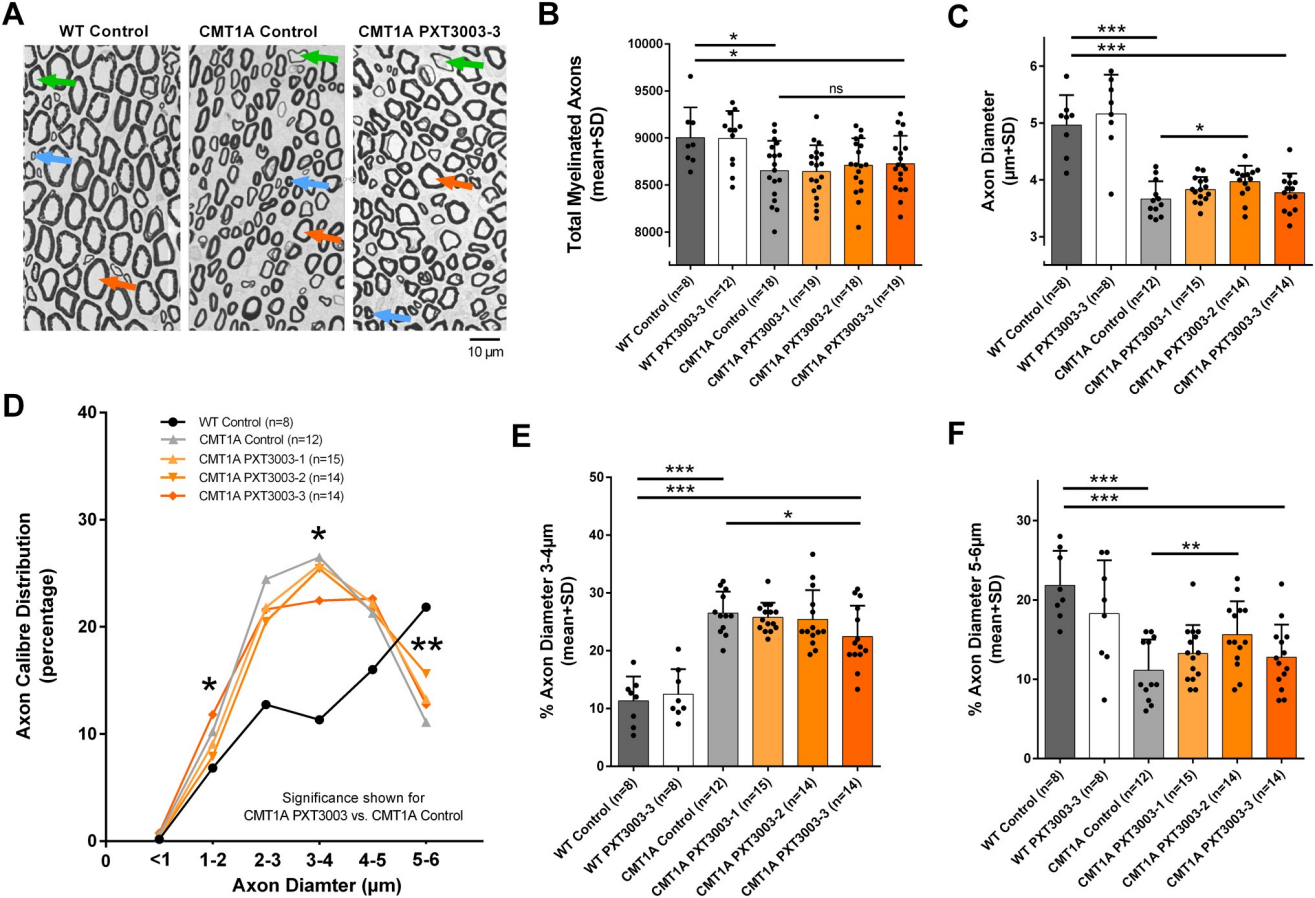
Shift towards large-calibre axons in the long-term observation. Histological analyses were performed on light microscopic level in the sciatic nerve as demonstrated in representative peripheral nerve cross sections. In contrast to WT controls, untreated CMT1A rats showed a loss of large-calibre axons (orange arrow) combined with a hypomyelination of predominantly large axons (green arrow) and a hypermyelination of predominantly small-calibre axons (blue arrow). A higher number of large-calibre axons appeared after PXT3003 treatment in CMT1A rats, without obvious differences between hypo- and hypermyelinated axons (A). Quantification of myelinated axons confirmed axonal loss in CMT1A controls not being affected by PXT3003 treatment (B; WT controls 9003±114, CMT1A controls 8654±74, CMT PXT3003-3 8727±68); however PXT3003 partially corrected the reduced mean axon diameter (C; WT controls 4.96±0.19, CMT1A controls 3.66±0.09, CMT PXT3003-2 3.97±0.08). More detailed analyses of axon calibre distribution confirmed an obvious loss of large-calibre axons in CMT1A rats, and a shift towards large-calibre axons after PXT3003 treatment (D). Mid- and large-calibre axons were corrected towards the wildtype situation as illustrated in more detail for 3–4μm and 5–6μm axons (E and F; WT controls 11.33±1.48, CMT1A controls 26.50±1.08, CMT PXT3003-3 22.44±1.43 and WT controls 21.83±1.54, CMT1A controls 11.11±1.12, CMT PXT3003-2 15.62±1.13, respectively). (ns = not significant, * = p<0.05, **p<0.01 and *** = p<0.001).

At trial end, total number of myelinated axons, the axon calibre size and myelin sheath thickness were quantified in peripheral nerves (representative photos shown in Fig 3A). In general, PXT3003 short-term treatment did not cause any adverse effects on histological level in wildtype rats (Fig 3B and 3C, and S1E and S1F Fig). As expected, we observed less myelinated axons in CMT1A compared to the wildtype controls, both in the sciatic nerve (approx. 5%) (Fig 3B) and the tibial nerve (approx. 5%) (S1D Fig) being in line with previous studies [27,32,34]. Early short-term treatment with PXT3003 did not significantly restore axonal loss in CMT1A rats compared to CMT1A controls, although a trend towards more myelinated axons may be assumed (Fig 3B and S1D Fig). However, a more detailed analysis revealed a significantly reduced mean axonal diameter in CMT1A controls compared to wildtypes, and this was significantly improved in PXT3003-2 short-term treated CMT1A rats (Fig 3C). We therefore then analysed the relative axon diameter distribution in the sciatic nerve and observed a significant loss of predominantly large-calibre axons above 5–6 μm in CMT1A controls compared to the wildtype situation (Fig 3F). On the other hand, there was a significant relative increase in mid-calibre axons of 3–4 μm (Fig 3E), and a trend for more small calibre axons of 1–2 μm in CMT1A controls. Interestingly, short-term treatment with PXT3003-2 and PXT3003-3 significantly improved this disturbed axon calibre distribution in CMT1A rats for large 5–6 μm (Fig 3D and 3F), mid 3–4 μm (Fig 3D and 3E) and small 1–2 μm axons (Fig 3D). We also measured myelin sheath thickness by g-ratio analysis; a g-ratio of 1 represents pathologically non-myelinated axons and g-ratios below 0.4 pathologically hypermyelinated axons. As described previously, wildtype controls showed a mean g-ratio of approximately 0.6, and CMT1A controls were characterised by a pathological hypermyelination of small-calibre axons combined with hypo- and non-myelinated axons (S1E and S1G Fig). However, myelin thickness in CMT1A rats was not altered by early short-term PXT3003 treatment after long-term observation (S1G–S1J Fig).

### Long-term effects after PXT3003 treatment on *Pmp22*, Schwann cell differentiation and biomarker expression

**Fig 4:**
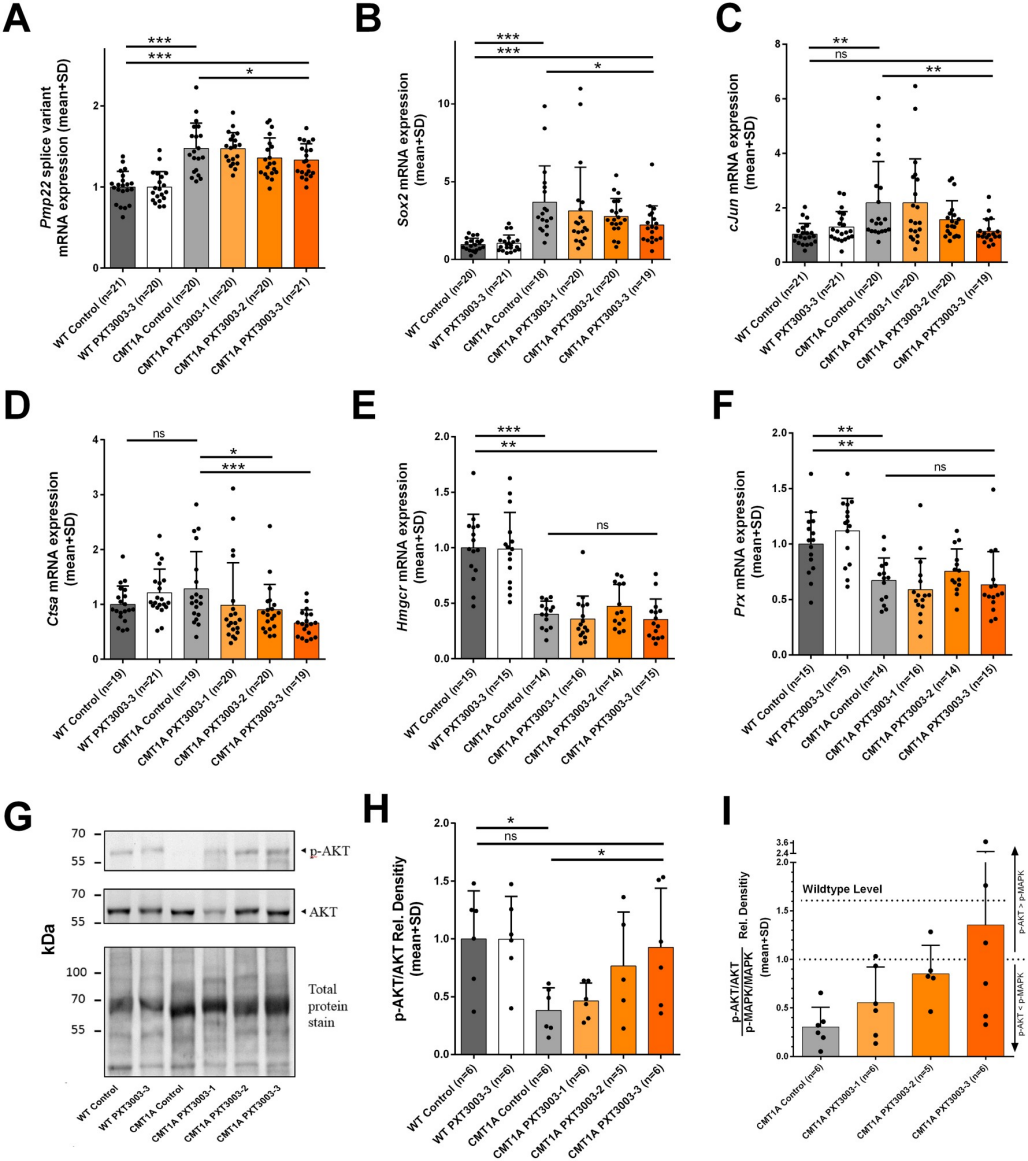
PXT3003 therapy acting on Pmp22 overexpression and downstream signalling in long-term at 12 weeks. Early short-term applied PXT3003 was in long-term effective on the downregulation of disease-causing *Pmp22* mRNA overexpression in CMT1A rats in the sciatic nerve (A; WT controls 1.00±0.04, CMT1A controls 1.48±0.07, CMT PXT3003-3 1.33±0.04; *Pmp22* splice variant normalised to C*yclophilin A)*. In the brachial plexus, mRNA expression of the differentiation marker *Sox2* (B; WT controls 1.00±0.09, CMT1A controls 3.68±0.55, CMT PXT3003-3 2.22±0.28; normalised to C*yclophili*n A and *Rplp0*) and *cJun* (C; WT controls 1.00±0.09, CMT1A controls 2.18±0.34, CMT PXT3003-3 1.14±0.10; normalised to C*yclophili*n A and *Rplp0*), as well as the biomarker *Ctsa* were influenced by PXT3003 in long-term observation (D; WT controls 1.00±0.08, CMT1A controls 1.28±0.16, CMT PXT3003-3 0.66±0.06; normalised to C*yclophili*n A and *Rplp0*). Downregulated *Hmgcr* and *Prx* mRNA expression in CMT1A rats were not affected by PXT3003 (E; WT controls 1.00±0.08, CMT1A controls 0.40±0.03, CMT PXT3003-3 0.35±0.05 and *F*; WT controls 1.00±0.07, CMT1A controls 0.67±0.05, CMT PXT3003-3 0.63±0.08; normalised to C*yclophili*n A and *Rplp0*). *Pmp22* downstream signalling was investigated by Western Blot analysis using antibodies against p-AKT/AKT and p-MAPK/MAPK (G). At the end of long-term observation, PXT3003 early short-term treatment restored the decreased p-AKT/AKT signalling in CMT1A rats (H; WT controls 1.00±0.17, CMT1A controls 0.38±0.08, CMT PXT3003-3 0.93±0.21) and dose-dependently corrected the disrupted ratio of p-AKT/AKT to p-MAPK/MAPK signalling towards the wildtype situation (I; CMT1A controls 0.30±0.08, CMT PXT3003-1 0.56±0.15, CMT PXT3003-2 0.85±0.13, CMT PXT3003-3 1.36±0.52), which is characterized by a higher relative amount of p-AKT versus p-MAPK. (ns = not significant, * = p<0.05, **p<0.01 and *** = p<0.001).

We confirmed the known significant approx. 1.6-fold *Pmp22* mRNA overexpression in the sciatic nerve of CMT1A controls compared to wildtypes (Fig 4A). We also detected a significant *Pmp22* mRNA overexpression in the more proximal brachial plexus in CMT1A rats, both for total *Pmp22* and splice variant mRNA (S1K and S1L Fig). PXT3003 therapy applied as short-term postnatally dose-dependently reduced *Pmp22* overexpression in CMT1A rats in the sciatic nerve for PXT3003-3 (Fig 4A) and in the brachial plexus when normalised to *Cyclophilin A* (S1K Fig). When normalised to *Mpz*, the downregulation was even more prominent reaching wildtype ratios (S1L Fig). Moreover, *Sox2* and *cJun*, known differentiation marker in the PNS, were also upregulated in the brachial plexus on the transcriptional level in CMT1A controls compared to the wildtype situation as described previously for sciatic nerve [27]. Both differentiation markers were dose-dependently corrected by PXT3003-3 (Fig 4B and 4C). *Ctsa*, a known biomarker originally identified from skin [15], was comparably expressed in CMT1A and wildtype controls but importantly, PXT3003-2 and PXT3003-3 short-term treated CMT1A rats showed long-lasting dose-dependent treatment effects on the transcriptional level (Fig 4D). *Hmgcr* and *Prx*, known lipid synthesis and myelin markers in the sciatic nerve [27], were downregulated also in the brachial plexus in CMT1A rats but not altered after short-term therapy with PXT3003 (Fig 4E and 4F). No effects of PXT3003 short-term treatment were observed on *Pmp22*, differentiation and biomarker mRNA expression in wildtype rats (Fig 4A–4F).

### Long-term impact on dysbalanced *Pmp22* downstream signalling pathways

Western Blotting analysis was performed to investigate the long-lasting effect of PXT3003 short-term treatment on known downstream signaling pathways in CMT1A rats. As described previously [27], the ratio of phosphorylated/non-phosphorylated PI3K-AKT was significantly reduced in CMT1A controls compared to wildtype controls (representative bands shown in Fig 4G). PXT3003 treatment in CMT1A rats increased this disturbed ratio in a dose-dependent manner reaching wildtype levels at the dosage PXT3003-3 (Fig 4H). The ratio of phosphorylated/non-phosphorylated MEK-ERK was not significantly regulated in 12 weeks aged rats, neither between CMT1A and wildtype controls nor after PXT3003 treatment. However, we observed a trend of a higher ratio of phosphorylated/non-phosphorylated MEK-ERK in CMT1A controls, which was decreased by PXT3003 treatment after long-term observation in single animals (S1M Fig). Hence, early postnatal short-term PXT3003 therapy corrected dose-dependently the disrupted ratio of p-AKT/AKT to p-MAPK/MAPK towards wildtype levels (Fig 4I).

## Discussion

In contrast to progesterone antagonists [32,34], the growth factor neuregulin [27] or, more recently, *Pmp22*-targeting antisense oligonucleotides [35,36], a preclinical early treatment trial in young CMT1A rats with PXT3003 is more easily translatable to young adults or even children with CMT1A due to its good safety profile. No adverse effects were observed in the wildtype or CMT1A rats treated with PXT3003 with regard to the weight, phenotype, electrophysiological parameters, histological abnormalities and molecular biology. Early short-term PXT3003 treatment during the peak time of myelination for approximately 2 weeks ameliorated the behavioural phenotype of CMT1A rats in a dose-dependent manner. First therapeutic effects of PXT3003 were already present in young 3 weeks aged CMT1A rats directly after the treatment period, when analysing the muscle strength of the forelimbs and the inclined plane performance. At this time point, PXT3003 effects were also present as dose-depended trends for muscle strength increase in the hindlimbs, possibly owing to longer axons. Until 12 weeks of age in CMT1A rats the levels of hindlimb muscle strength and inclined plane performance continuously increased reaching wildtype levels after PXT3003 short-term treatment. We note that the drug effect disappeared for the forelimbs until study end. However, forelimb muscle strength is limited for the long-term monitoring of drug effects since motor strength development—contrary to the hindlimbs—reaches wildtype levels in CMT1A controls in long-term [32]. Therefore, drug effects in the forelimbs may be compensated by model limitations.

The effect of PXT3003 early short-term treatment in CMT1A rats during the critical time window of Schwann cell differentiation is reminiscent of the effect of rhNRG treatment regimen [27]. Both therapeutic approaches successfully restored the phenotype performance of CMT1A rats towards wildtype level without the need for chronic drug application. rhNRG treatment in CMT1A rats corrected the dysdifferentiation status of Schwann cells, however without effects on *Pmp22* levels itself. In contrast, we here observed that PXT3003 short-term treatment downregulated *Pmp22* overexpression, a potential target for other preclinical trials in CMT1A rats [15,34,35,37], as demonstrated in two different peripheral nerves. We also observed a long-lasting improved misbalance in the PI3K-AKT and MEK-ERK downstream signaling pathway in sciatic nerves of CMT1A rats after PXT3003 short-term treatment. In contrast to *Pmp22* transgenic rats, mice lacking one copy of *Pmp22* [38], displayed increased PI3K-AKT signaling in sciatic nerve, which suggests a direct effect of cellular *Pmp22* levels on PI3K-AKT signaling [27]. This argues that early short-term PXT3003 treatment downregulated *Pmp22* mRNA overexpression, improved the downstream PI3K-AKT / MEK-ERK signaling pathway, and thereby influenced the differentiation status of Schwann cells in long-term as shown by a dose dependent effect on the dedifferentiation markers *cJun* and *Sox2*. This may, in turn, enable an improved long-lasting axonal support function of Schwann cells in CMT1A rats [39].

Surprisingly, we did not observe an altered myelin sheath thickness in peripheral nerve of PXT3003 treated CMT1A rats, and in line PXT3003 therapy had no effects on the major lipid synthesizing *Hmgcr* or the myelin marker *Prx* expression. The loss of myelinated axons was also not restored in long-term. Obviously, neither the number of myelinated axons nor the myelin deficits contributed to the restored long-term phenotype performance in CMT1A rats. On the other hand, a time delay between phenotypical and histological effects may be assumed, and treatment effects may become more evident after longer early treatment regimens as already reported for the long-term application of PXT3003 in adult CMT1A rats over 4 months [24].

However, a more detailed analysis of axon calibre sizes in peripheral nerve identified a disturbed axon calibre distribution towards small-calibre axons in CMT1A rats, which reflects a relative loss of large motor axons in CMT1A, and PXT3003 treatment in CMT1A rats shifted the disturbed axon calibre distribution towards wildtype levels, similar to previous studies [32]. In addition, electrophysiological analysis identified improved distal motor latencies (DML) after PXT3003 short-term treatment in CMT1A rats compared to CMT1A controls which correlated with the improved phenotype performance after long-term observation, similar to the exploratory clinical phase 2 study in CMT1A patients [25,26]. The DML is thought to include saltatory conduction speed, but also terminal transmission of the nerve into the muscle via the neuromuscular junction (NMJ). Altered NMJ innervation has been described for the Connexin32 mouse model of human CMTX, but not for CMT1A [40]. Therefore, PXT3003 short-term treatment in CMT1A rats may support functional axonal integrity of predominantly larger calibre motor axons uncoupled from dysmyelination and may functionally enhance terminal muscle innervation at the NMJ, both contributing to rescued long-term muscle strength.

We conclude that short-term treatment with PXT3003 during early development partially prevents the long-term phenotypical manifestations in CMT1A rats, similar to levels after a later, but longer treatment [24]. Long-term effects can be explained by an improved Schwann cell differentiation leading to enhanced axonal support of predominantly large-caliber axons, and possibly a proper terminal transmission at the NMJ. Since we observed increasing phenotype effects over time reaching wildtype levels at study end, one may argue for positive long-term effects beyond the age of 12 weeks. However, the histological deficits were not dramatically improved and longer effects until late adulthood must be investigated in a separate study. If translated to CMT1A patients, treatment initiation should preferentially begin as early as possible (e.g. in childhood), in order to slow down or even prevent the progression of the disease.

## Supporting information

S1 Fig Complementary phenotypical, histological and molecular observations. At treatment start, i.e. before the manifestation of obvious phenotype deficits, body weights did not differ between 6 days aged wildtype and CMT1A controls, and all CMT1A PXT3003 groups showed comparable body weights representing a well-balanced randomisation (A; WT controls 31.79±0.75, CMT1A controls 29.61±0.99, CMT PXT3003-3 29.51±1.04). PXT-3003 did not influenced body weight development throughout the observational period until 12 weeks of age (B; WT controls 475.3±9.42, CMT1A controls 439.9±8.56, CMT PXT3003-3 436.2±7.61). PXT3003 increased forelimb muscle strength in young CMT1A rats but effects disappeared in long-term observation (C). Quantification of myelinated axons was also performed in the tibial nerve and confirmed axonal loss in CMT1A controls not being affected by PXT3003 treatment (D; WT controls 3234±31, CMT1A controls 3107±42, CMT PXT3003-3 3137±51). Myelin sheath thickness analysis confirmed the presence of non-myelinated axons solely in CMT1A rats (g-ratio = 1) and a shift towards hypomyelinated large-calibre and hypermyelinated small-calibre axons in CMT1A rats (E and G), both not being affected by PXT3003 treatment (G-J). PXT3003 downregulated Pmp22 mRNA overexpression in CMT1A rats in the plexus brachialis when normalised to both, Cyclophilin A (K; Pmp22 splice variant, WT controls 1.00±0.05, CMT1A controls 1.53±0.09, CMT PXT3003-3 1.20±0.08) and Mpz (L; total Pmp22, WT controls 1.00±0.08, CMT1A controls 1.94±0.28, CMT PXT3003-3 1.09±0.20). p-MAPK/MAPK signalling was neither significantly regulated between WT and CMT1A controls nor after PXT3003 treatment in WT and CMT1A rats at the age of 12 weeks although strong trends were observed (M; WT controls 1.00±0.27, CMT1A controls 1.74±0.62, CMT PXT3003-3 0.88±0.12). (ns = not significant, * = p<0.05, **p<0.01 and *** = p<0.001).
(TIF)
